# Supplementary material for: Nascent RHOH acts as a molecular brake on actomyosin-mediated effector functions of inflammatory neutrophils
Source: PLoS Biol. 2022 Sep 15;20(9):e3001794. doi: 10.1371/journal.pbio.3001794 (PMC9514642; doi:10.1371/journal.pbio.3001794)
Supplement: S1 Fig — (A) Heatmap showing fold change of RHOH gene expression in human neutrophils treated with GM-CSF at different time points (compared with freshly isolated neutrophils). The underlying data can be found in S1 Data. (B) Violin plots showing RHOH gene expression in cell populations identified in COVID-19 patients. Mono, monocytes; NK, natural killer cells; Macro, macrophages; Epi, epithelial cells; Neu, neutrophils, shown in black box (□); DC, dendritic cells; Plasma, plasma B cells; Mega, megakaryocytes. The underlying data can be found in https://www.ncbi.nlm.nih.gov/geo/query/acc.cgi?acc=GSE158055. (C) Violin plots showing the expression of Rhoh in major immune cell subsets identified in MC38 tumors. Neutrophils are shown in black box (□). The underlying data can be found in https://www.ebi.ac.uk/biostudies/arrayexpress/studies/E-MTAB-8832. (DOCX) [file pbio.3001794.s001.docx]

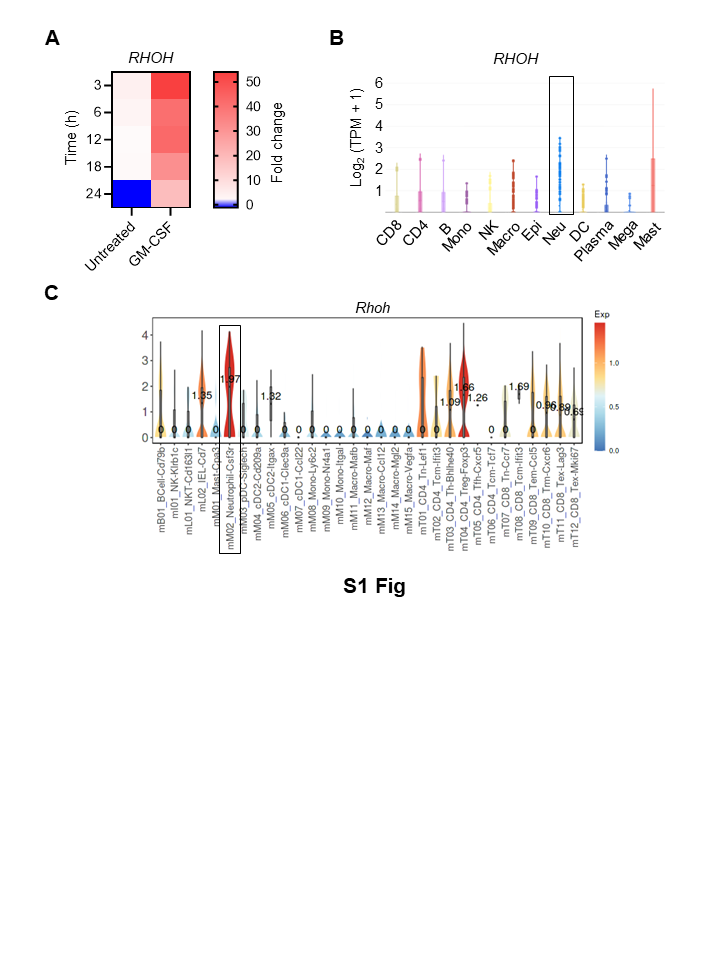


**S1 Fig. *RHOH* expression is upregulated in neutrophils under different inflammatory conditions. A** Heatmap showing fold change of *RHOH* gene expression in human neutrophils treated with GM-CSF at different time points (compared with freshly isolated neutrophils). The underlying data can be found in S1 Data. **B** Violin plots showing *RHOH* gene expression in cell populations identified in COVID-19 patients. Mono, monocytes; NK, natural killer cells; Macro, macrophages; Epi, epithelial cells; Neu, neutrophils, shown in black box (□); DC, dendritic cells; Plasma, plasma B cells; Mega, megakaryocytes. The underlying data can be found in <https://www.ncbi.nlm.nih.gov/geo/query/acc.cgi?acc=GSE158055>**. C** Violin plots showing the expression of *Rhoh* in major immune cell subsets identified in MC38 tumors. Neutrophils are shown in black box (□). The underlying data can be found in <https://www.ebi.ac.uk/biostudies/arrayexpress/studies/E-MTAB-8832>.
