# Supplementary material for: Nascent RHOH acts as a molecular brake on actomyosin-mediated effector functions of inflammatory neutrophils
Source: PLoS Biol. 2022 Sep 15;20(9):e3001794. doi: 10.1371/journal.pbio.3001794 (PMC9514642; doi:10.1371/journal.pbio.3001794)
Supplement: S2 Fig — (A) Protein lysates of human neutrophils isolated from healthy donors treated with medium or serum from HD and CF patients (6 h) were analyzed by immunoblot for RHOH protein expression. (B–D) Pretreated human neutrophils with medium or serum from HD or CF patients (6 h) were subsequently activated with the indicated stimuli. (B) Neutrophil degranulation was determined using the surrogate marker CD63 by flow cytometry (sample number: 1, 2, 3, 4). (C) Quantification of released dsDNA in the culture supernatants (sample number: 1, 2, 3). (D) NETs were visualized by the colocalization of NE (green) with released dsDNA (PI, red) using confocal microscopy (sample number: 1, 2, 3). Scale bars, 10 μm. (D) Data are representative of 3 independent experiments. (B, C) Values are means ± SD. Two-way ANOVA with Šídák’s multiple comparisons test was applied. The underlying data for S2B and S2C Fig can be found in S1 Data. The underlying data for S2A Fig can be found in S1 Raw images. CF, cystic fibrosis; dsDNA, double-stranded DNA; HD, healthy donor; NE, neutrophil elastase; NET, neutrophil extracellular trap. (DOCX) [file pbio.3001794.s002.docx]

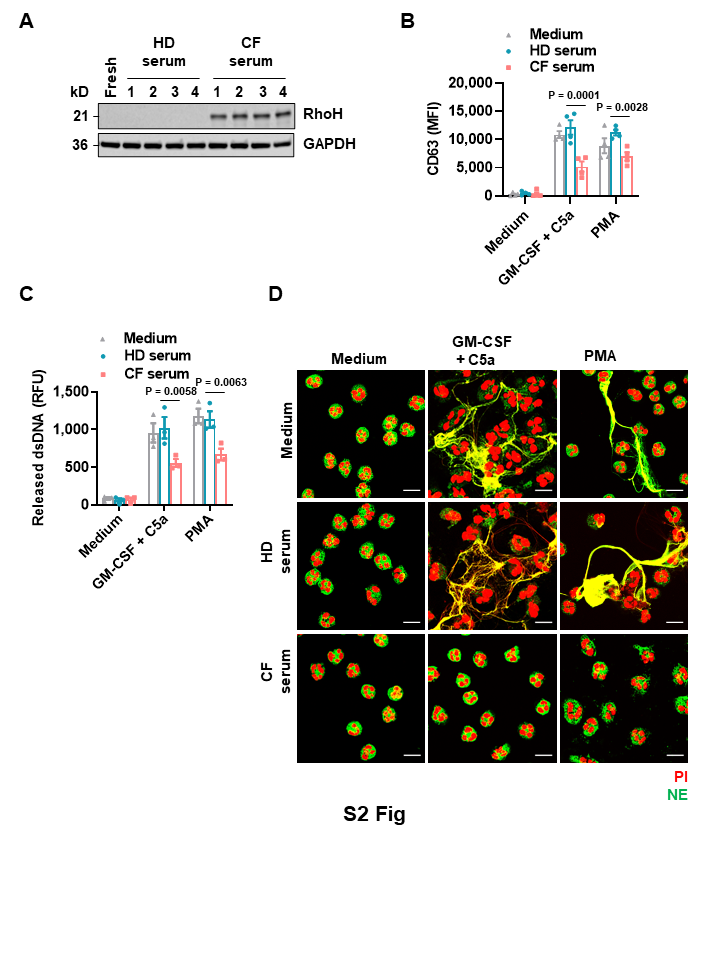


**S2 Fig. Human neutrophils stimulated with serum from CF patients show similarity with CF neutrophils.** **A** Protein lysates of human neutrophils isolated from healthy donors treated with medium or serum from HD and CF patients (6 h) were analyzed by immunoblot for RHOH protein expression. **B-D** Pretreated human neutrophils with medium or serum from HD or CF patients (6 h) were subsequently activated with the indicated stimuli. **B** Neutrophil degranulation was determined using the surrogate marker CD63 by flow cytometry (sample number: 1, 2, 3, 4). **C** Quantification of released dsDNA in the culture supernatants (sample number: 1, 2, 3). **D** NETs were visualized by the colocalization of neutrophil elastase (NE, green) with released dsDNA (PI, red) using confocal microscopy (sample number: 1, 2, 3). Scale bars, 10 μm. **D** Data are representative of three independent experiments. **B, C** Values are means ± SD. Two-way ANOVA with Šídák’s multiple comparisons test was applied. The underlying data for S2B and S2C Fig can be found in S1 Data. The underlying data for S2A Fig can be found in S1 Raw Images.
