# Supplementary material for: Nascent RHOH acts as a molecular brake on actomyosin-mediated effector functions of inflammatory neutrophils
Source: PLoS Biol. 2022 Sep 15;20(9):e3001794. doi: 10.1371/journal.pbio.3001794 (PMC9514642; doi:10.1371/journal.pbio.3001794)
Supplement: S3 Fig — (A) Representative microscopic images of neutrophils freshly isolated from WT and Rhoh-/- mice. (B) The mRNA expression of Rhoh in WT and Rhoh-/- neutrophils was quantified by Q-PCR. (C, D) Freshly isolated neutrophils from WT and Rhoh-/- mice were pretreated with GM-CSF for indicated time followed by activation with LPS or PMA for 15 min. (C) Extracellular DNA fibers were stained with MitoSOX Red and the nucleus with Hoechst 33342 and analyzed by confocal microscopy. Scale bars, 10 μm. Data are representative of three independent experiments. (D) Quantification of released dsDNA in the culture supernatants. Unpaired 2-tailed Student t test (B) or 2-way ANOVA with Tukey’s multiple comparisons test (D) was applied. Values are means ± SD. The underlying data for S3B and S3D Fig can be found in S1 Data. dsDNA, double-stranded DNA; GM-CSF, granulocyte/macrophage colony-stimulating factor; NET, neutrophil extracellular trap. (DOCX) [file pbio.3001794.s003.docx]

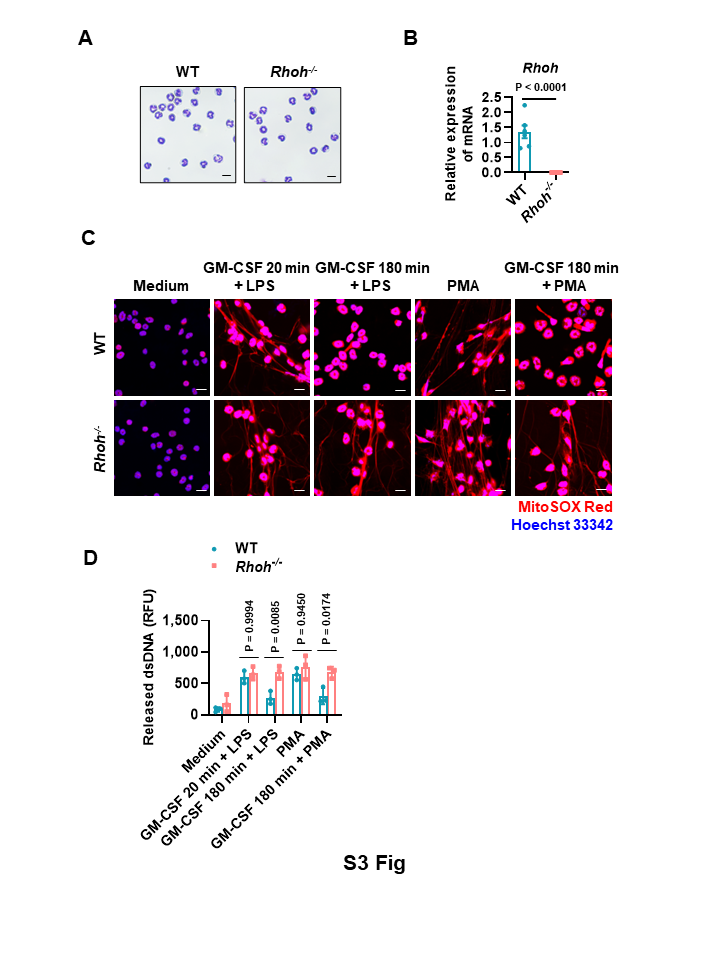


**S3** **Fig. Induction of RhoH expression by GM-CSF stimulation suppresses NET formation.** **A** Representative microscopic images of neutrophils freshly isolated from WT and *Rhoh^-/-^* mice. **B** The mRNA expression of *Rhoh* in WT and *Rhoh^-/-^* neutrophils was quantified by Q-PCR. **C, D** Freshly isolated neutrophils from WT and *Rhoh^-/-^* mice were pretreated with GM-CSF for indicated time followed by activation with LPS or PMA for 15 min. **C** Extracellular DNA fibers were stained with MitoSOX Red and the nucleus with Hoechst 33342 and analyzed by confocal microscopy. Scale bars, 10 μm. Data are representative of three independent experiments. **D** Quantification of released dsDNA in the culture supernatants. Unpaired two-tailed Student’s *t*-test (**B**) or two-way ANOVA with Tukey’s multiple comparisons test (**D**) was applied. Values are means ± SD. The underlying data for S3B and S3D Fig can be found in S1 Data.
