# Supplementary material for: Nascent RHOH acts as a molecular brake on actomyosin-mediated effector functions of inflammatory neutrophils
Source: PLoS Biol. 2022 Sep 15;20(9):e3001794. doi: 10.1371/journal.pbio.3001794 (PMC9514642; doi:10.1371/journal.pbio.3001794)
Supplement: S4 Fig — (A) Representative images showing nuclear morphology of HoxB8 cells before and after differentiation into mature neutrophils. Scale bar, 10 μm. (B) Cell surface expression of Ly6G of HoxB8 cells before and after 5-day differentiation was analyzed by flow cytometry. Data are representative of 3 independent experiments. (DOCX) [file pbio.3001794.s004.docx]

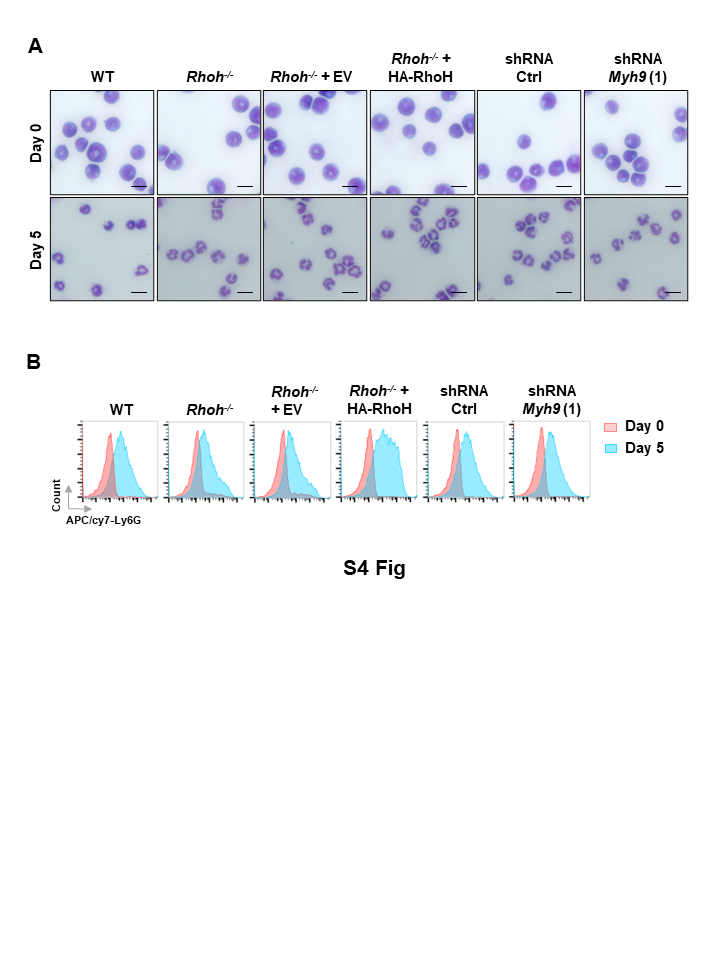


**S4** **Fig. HoxB8 neutrophils are characterized by nuclear morphology and flow cytometry. A** Representative images showing nuclear morphology of HoxB8 cells before and after differentiation into mature neutrophils. Scale bar, 10 μm. **B** Cell surface expression of Ly6G of HoxB8 cells before and after 5-days differentiation was analyzed by flow cytometry. Data are representative of three independent experiments.
