# Supplementary material for: Nascent RHOH acts as a molecular brake on actomyosin-mediated effector functions of inflammatory neutrophils
Source: PLoS Biol. 2022 Sep 15;20(9):e3001794. doi: 10.1371/journal.pbio.3001794 (PMC9514642; doi:10.1371/journal.pbio.3001794)
Supplement: S5 Fig — (A) Protein expression of NMHC IIA in mature HoxB8 neutrophils treated with control (Ctrl) or the second Myh9 shRNA was analyzed by immunoblotting. (B) Neutrophil degranulation was determined by flow cytometry. (C) Extracellular DNA fibers were analyzed by confocal microscopy. Scale bars, 10 μm. At least 3 independent experiments were performed. (D) Quantification of released dsDNA in culture supernatants. (B, D) Values are means ± SD. (B, D) Two-way ANOVA with Šídák’s multiple comparisons test. The underlying data for S5B and S5D Fig can be found in S1 Data. The underlying data for S5A Fig can be found in S1 Raw images. (DOCX) [file pbio.3001794.s005.docx]

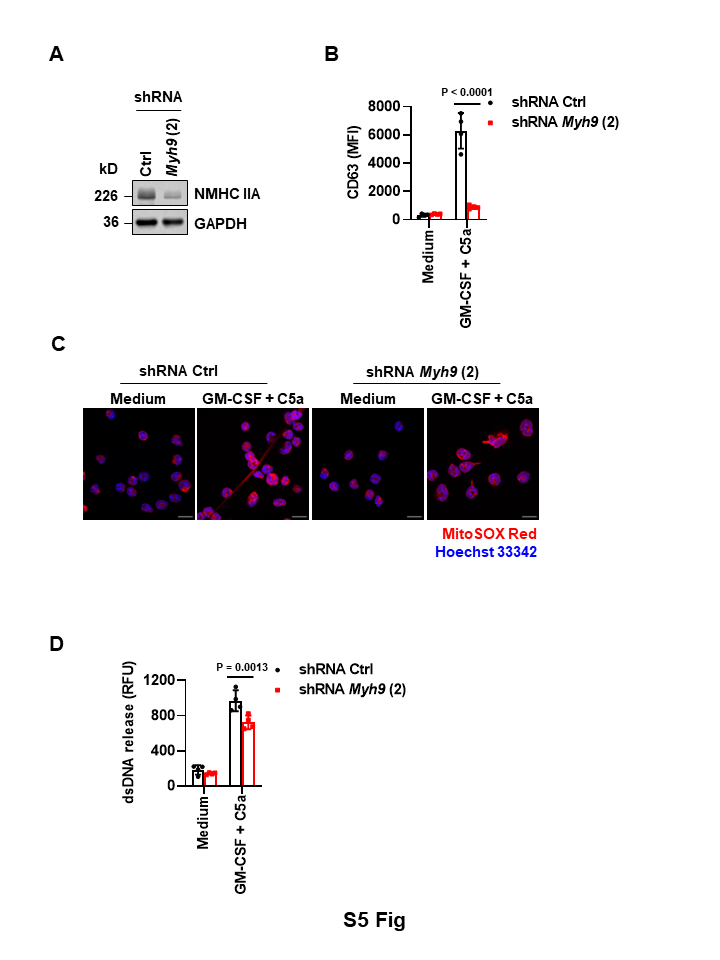


**S5 Fig. Reduced NMHC IIA expression by a second shRNA targeting *Myh9* decreases NET formation.** **A** Protein expression of NMHC IIA in mature HoxB8 neutrophils treated with control (Ctrl) or the second Myh9 shRNA was analyzed by immunoblotting. **B** Neutrophil degranulation was determined by flow cytometry. **C** Extracellular DNA fibers were analyzed by confocal microscopy. Scale bars, 10 μm. At least three independent experiments were performed. **D** Quantification of released dsDNA in culture supernatants. B, D Values are means ± SD. B, D Two-way ANOVA with Šídák’s multiple comparisons test. The underlying data for S5B and S5D Fig can be found in S1 Data. The underlying data for S5A Fig can be found in S1 Raw Images.
