# Supplementary material for: Nascent RHOH acts as a molecular brake on actomyosin-mediated effector functions of inflammatory neutrophils
Source: PLoS Biol. 2022 Sep 15;20(9):e3001794. doi: 10.1371/journal.pbio.3001794 (PMC9514642; doi:10.1371/journal.pbio.3001794)
Supplement: S6 Fig — (A–C) Human neutrophils were treated with vehicle control or myosin IIA inhibitors for 30 min followed by the indicated stimulation. (A) F-actin distribution was analyzed by confocal microscopy (upper part). Scale bars, 10 μm. Cell area and F-actin intensity were quantified by using Imaris software (lower part). A total of 24 images (each containing 8–15 cells) from 3 independent experiments were included for each condition. (B) Phosphorylation of myosin IIA was analyzed by immunoblotting. (C) ROS activity was assessed using DHR 123 dye by flow cytometry. Data are representative of 3 independent experiments. Values represent means ± SD. One-way ANOVA with Dunnett’s multiple comparisons test was applied. The underlying data for S6A and S6C Fig can be found in S1 Data. The underlying data for S6B Fig can be found in S1 Raw images. (DOCX) [file pbio.3001794.s006.docx]

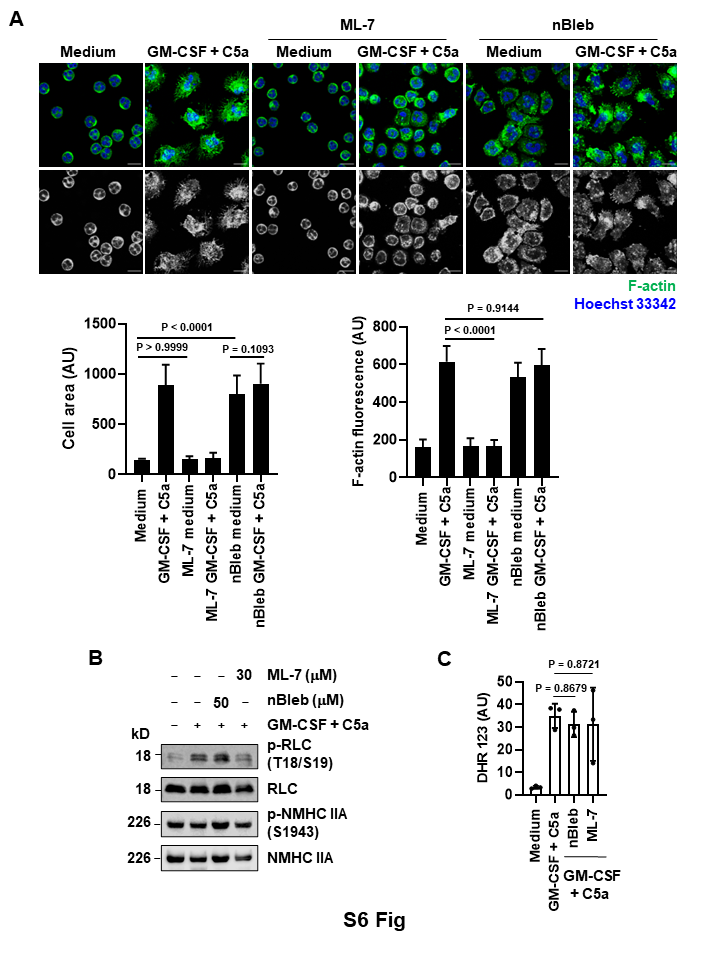


**S6 Fig. Effects of myosin IIA inhibitors on human neutrophils. A-C** Human neutrophils were treated with vehicle control or myosin IIA inhibitors for 30 min followed by the indicated stimulation. **A** F-actin distribution was analyzed by confocal microscopy (upper part). Scale bars, 10 μm. Cell area and F-actin intensity were quantified by using Imaris software (lower part). 24 images (each containing 8-15 cells) from three independent experiments were included for each condition. **B** Phosphorylation of myosin IIA were analyzed by immunoblotting. **C** ROS activity was assessed using DHR 123 dye by flow cytometry. Data are representative of three independent experiments. Values represent means ± SD. One-way ANOVA with Dunnett's multiple comparisons test was applied. The underlying data for S6A and S6C Fig can be found in S1 Data. The underlying data for S6B Fig can be found in S1 Raw Images.
