# Supplementary material for: Nascent RHOH acts as a molecular brake on actomyosin-mediated effector functions of inflammatory neutrophils
Source: PLoS Biol. 2022 Sep 15;20(9):e3001794. doi: 10.1371/journal.pbio.3001794 (PMC9514642; doi:10.1371/journal.pbio.3001794)
Supplement: S7 Fig — (A) The PNL from activated neutrophils expressing HA-RhoH or the EV was separated and collected equally into 1–10 fractions followed by immunoblot analysis. (B) Immunoblot analysis of NMHC IIA in cytosolic (C) and mitochondrial (M) fractions from activated neutrophils expressing HA-RhoH or EV. Data are representative of 3 independent experiments. The underlying data for S7A and S7B Fig can be found in S1 Raw images. EV, empty vector; NMHC IIA, non-muscle myosin heavy chain IIA; PNL, postnuclear lysate. (DOCX) [file pbio.3001794.s007.docx]

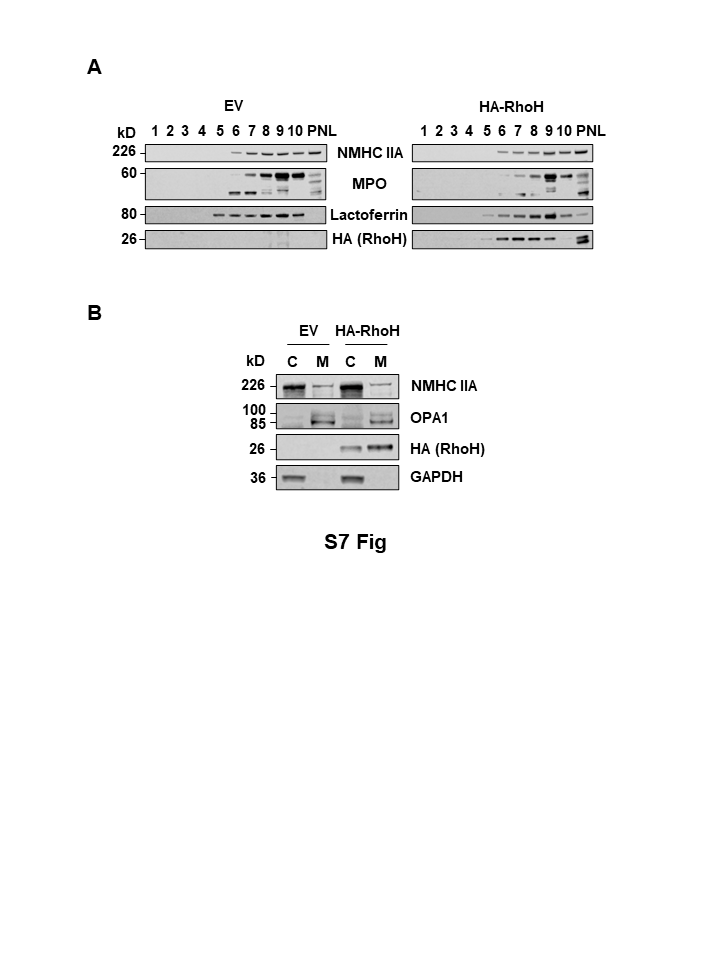


**S7** **Fig. Re-expression of RhoH does not affect the localization of NMHC IIA in neutrophil granules and mitochondria. A** The post-nuclear lysate (PNL) from activated neutrophils expressing HA-RhoH or the empty vector (EV) was separated and collected equally into 1-10 fractions followed by immunoblot analysis. **B** Immunoblot analysis of NMHC IIA in cytosolic (C) and mitochondrial (M) fractions from activated neutrophils expressing HA-RhoH or EV. Data are representative of three independent experiments. The underlying data for S7A and S7B Fig can be found in S1 Raw Images.
