# Supplementary material for: Nascent RHOH acts as a molecular brake on actomyosin-mediated effector functions of inflammatory neutrophils
Source: PLoS Biol. 2022 Sep 15;20(9):e3001794. doi: 10.1371/journal.pbio.3001794 (PMC9514642; doi:10.1371/journal.pbio.3001794)
Supplement: S8 Fig — (A) The mRNA expression of Rhoh in neutrophils expressing WT or mutated HA-RhoH or the corresponding EV was quantified by Q-PCR. Values are means ± SD. One-way ANOVA with Dunnett’s multiple comparisons test was applied. (B) Neutrophils expressing HA-RhoHY33F were treated with MG132 or Baf A1 for 4 h followed by immunoblot analysis. MG132 (10 μm) was used as a proteasome inhibitor; Baf A1 (250 nM) was used as a lysosomal enzyme inhibitor. Rac1, Rho GDI, and actin were used as loading control. Values represent means ± SD from 3 independent experiments. One-way ANOVA with Tukey’s multiple comparisons test was applied. The underlying numerical data for S8A and S8B Fig can be found in S1 Data. The uncropped immunoblots for S8B Fig can be found in S1 Raw images. EV, empty vector. (DOCX) [file pbio.3001794.s008.docx]

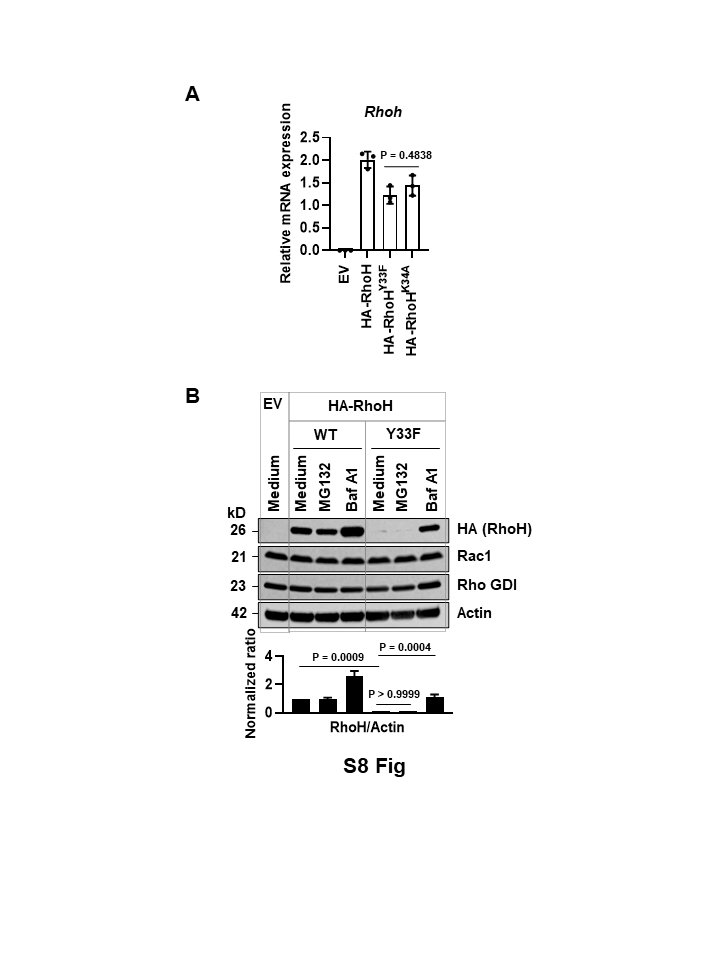


**S8** **Fig. RhoH mutation at tyrosine 33 (RhoH^Y33F^) results in lysosomal degradation of RhoH protein. A** The mRNA expression of *Rhoh* in neutrophils expressing WT or mutated HA-RhoH or the corresponding empty vector (EV) was quantified by Q-PCR. Values are means ± SD. One-way ANOVA with Dunnett's multiple comparisons test was applied. **B** Neutrophils expressing HA-RhoH^Y33F^ were treated with MG132 or Baf A1 for 4 h followed by immunoblot analysis. MG132 (10 μM) was used as a proteasome inhibitor; Baf A1 (250 nM) was used as a lysosomal enzyme inhibitor. Rac1, Rho GDI and actin were used as loading control. Values represent means ± SD from three independent experiments. One-way ANOVA with Tukey’s multiple comparisons test was applied. The underlying numerical data for S8A and S8B Fig can be found in S1 Data. The uncropped immunoblots for S8B Fig can be found in S1 Raw Images.
