# Supplementary material for: Nascent RHOH acts as a molecular brake on actomyosin-mediated effector functions of inflammatory neutrophils
Source: PLoS Biol. 2022 Sep 15;20(9):e3001794. doi: 10.1371/journal.pbio.3001794 (PMC9514642; doi:10.1371/journal.pbio.3001794)
Supplement: S9 Fig — (A–D) The 5-day differentiated HoxB8 neutrophils expressing HA-RhoH or EV were treated as indicated. (A) Microtubule assembly in these cells was analyzed by confocal microscopy (left). Scale bars, 10 μm. Quantification of microtubule was performed by automated analysis of microscopic images using Imaris software (right). Values are means ± SD. Two-way ANOVA with Tukey’s multiple comparisons test was applied. (B) ROS activity was assessed by flow cytometry. (C, D) The levels of GTP-bound and total Cdc42 protein (C), GTP-bound and total RhoA protein (D), were compared using effector pulldown assay, followed by immunoblotting. GTPγS was used as a positive control, GDP as a negative control. All data are representative of 3 independent experiments. The underlying data for S9A and S9B Fig can be found in S1 Data. The underlying data for S9C and S9D Fig can be found in S1 Raw images. EV, empty vector. (DOCX) [file pbio.3001794.s009.docx]

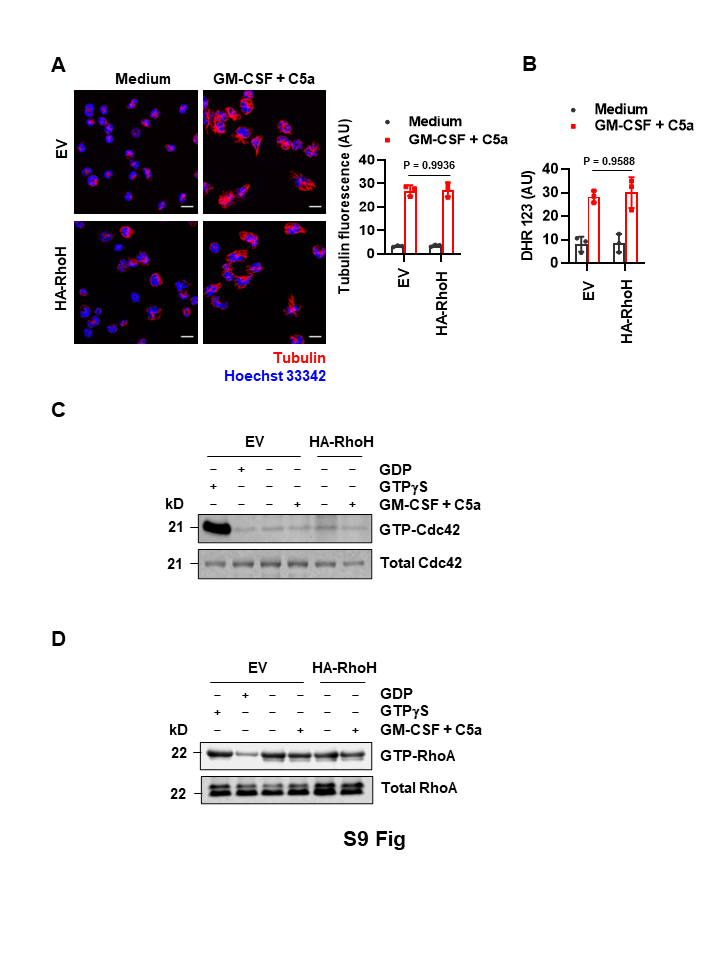


**S9 Fig. RhoH is not involved in microtubule rearrangement and does not interfere with Cdc42 and RhoA activity. A-D** 5-days differentiated HoxB8 neutrophils expressing HA-RhoH or empty vector (EV) were treated as indicated. **A** Microtubule assembly in these cells was analyzed by confocal microscopy (left). Scale bars, 10 μm. Quantification of microtubule was performed by automated analysis of microscopic images using Imaris software (right). Values are means ± SD. Two-way ANOVA with Tukey’s multiple comparisons test was applied. **B** ROS activity was assessed by flow cytometry. **C, D** The levels of GTP-bound and total Cdc42 protein (**C**), GTP-bound and total RhoA protein (**D**), were compared using effector pulldown assay, followed by immunoblotting. GTPγS was used as a positive control, GDP as a negative control. All data are representative of three independent experiments. The underlying data for S9A and S9B Fig can be found in S1 Data. The underlying data for S9C and S9D Fig can be found in S1 Raw Images.
