# Supplementary material for: Nascent RHOH acts as a molecular brake on actomyosin-mediated effector functions of inflammatory neutrophils
Source: PLoS Biol. 2022 Sep 15;20(9):e3001794. doi: 10.1371/journal.pbio.3001794 (PMC9514642; doi:10.1371/journal.pbio.3001794)

Fig 1B

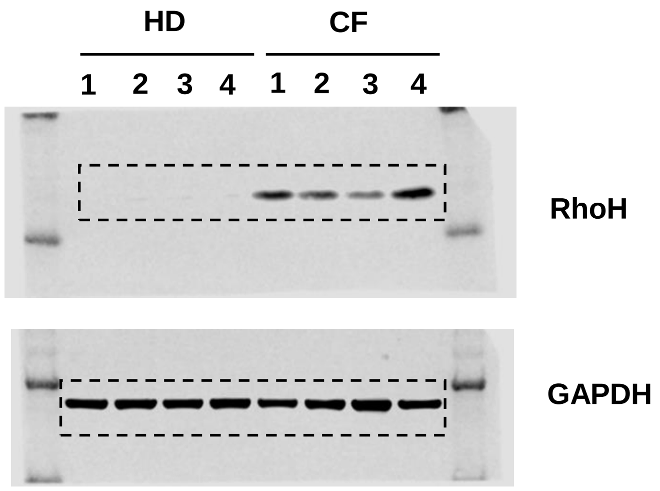

Fig 2A

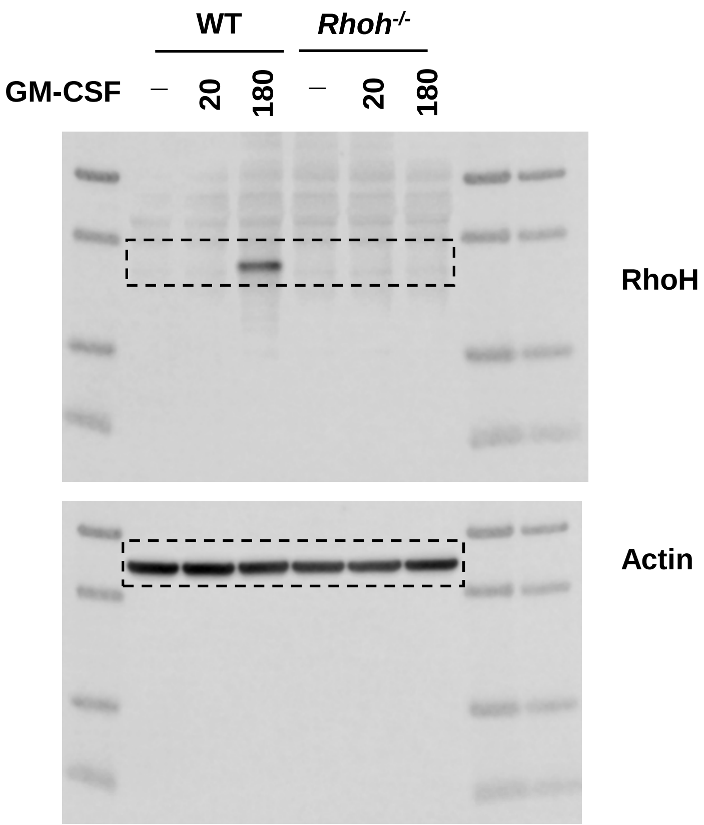
WT
Rhoh-/-
GM-CSF
—
20
180
—
20
180
RhoH
Actin

Fig 2E

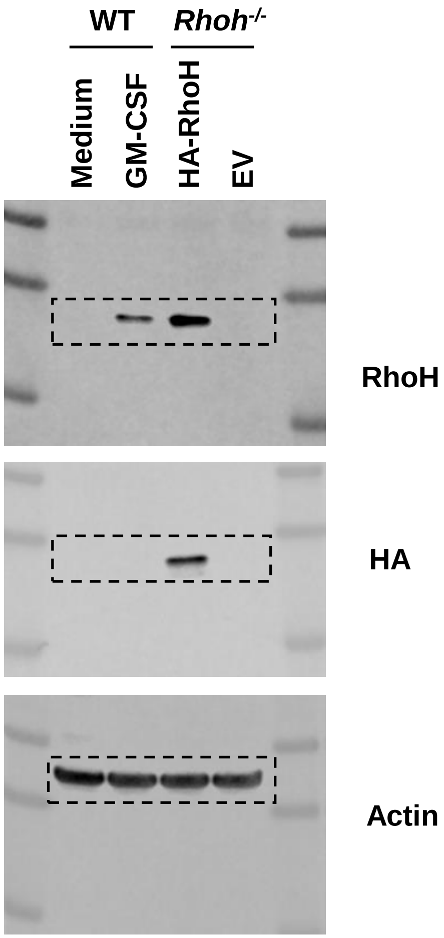
WT
Rhoh-/-
Medium
GM-CSF
HA-RhoH
EV
RhoH
HA
Actin

Fig 3A

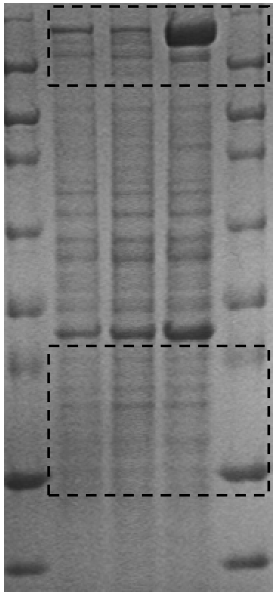

Fig 3B

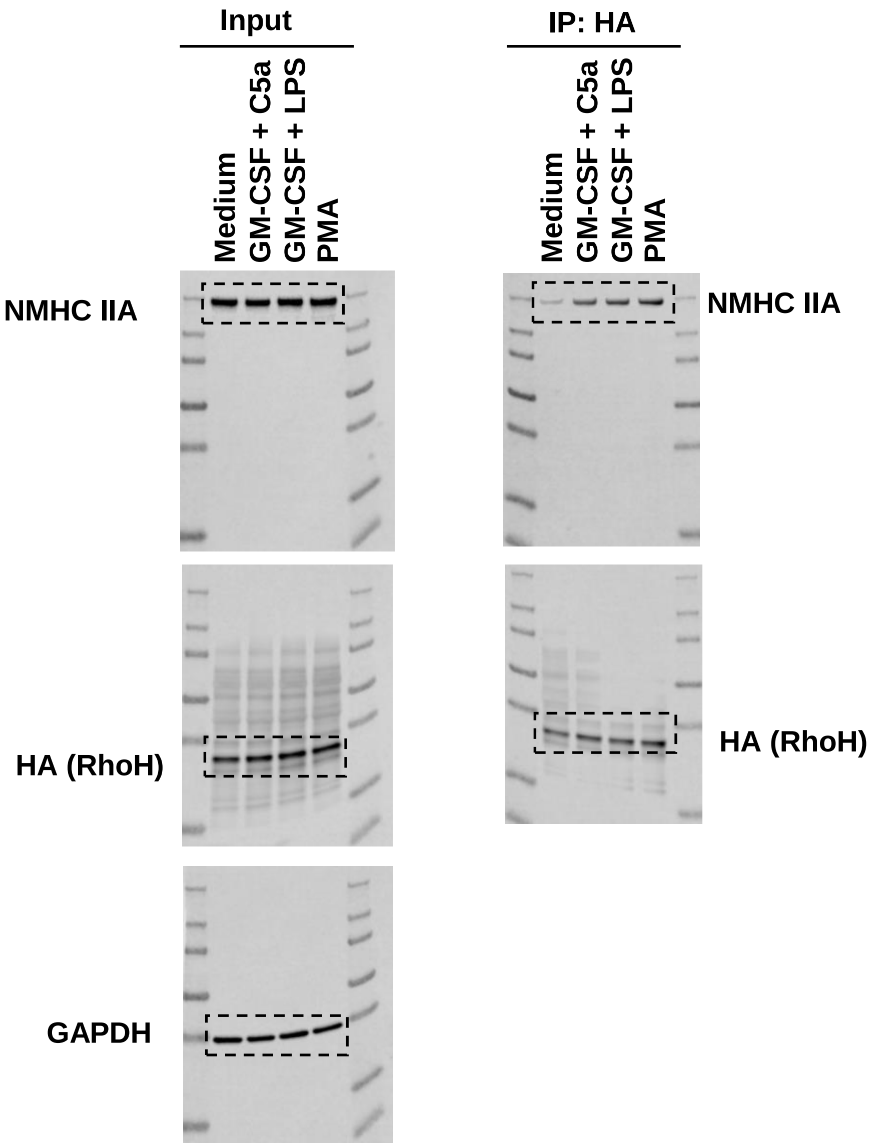
Input
IP: HA
Medium
GM-CSF + C5a
GM-CSF + LPS
PMA
NMHC IIA
HA (RhoH)
GAPDH

Fig 3C

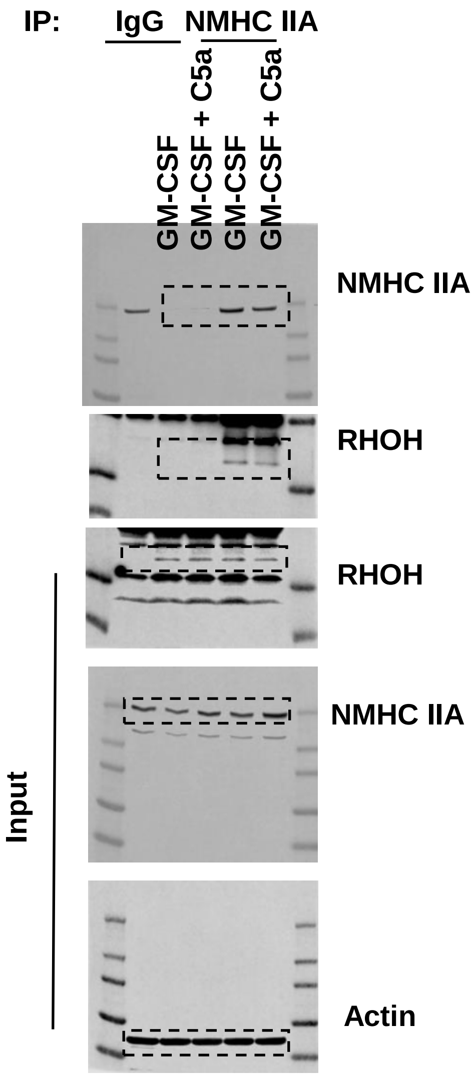
IP:
IgG
NMHC IIA
GM-CSF
GM-CSF + C5a
GM-CSF
GM-CSF + C5a
NMHC IIA
RHOH
RHOH
NMHC IIA
Actin
Input

Fig 3G

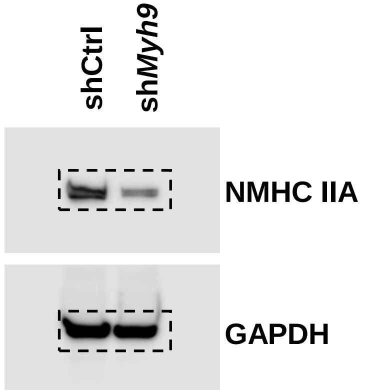
shCtrl
sh*Myh9*
NMHC IIA
GAPDH

Fig 4A

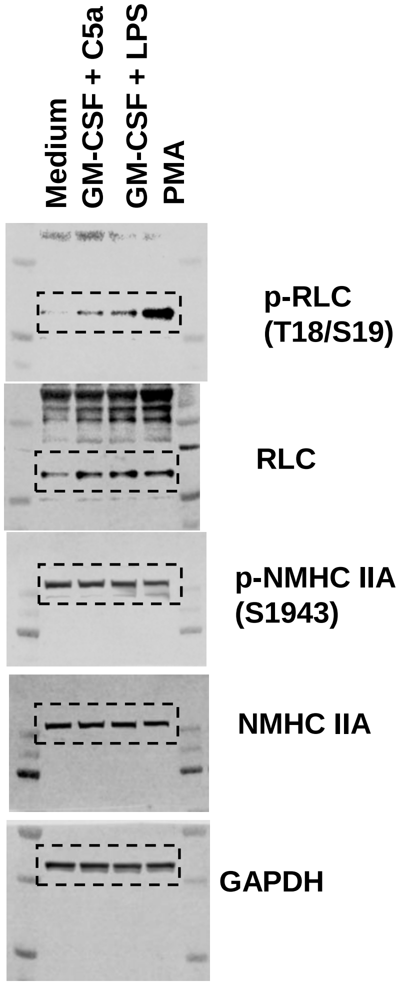

Medium
GM-CSF + C5a
GM-CSF + LPS
PMA
p-RLC
(T18/S19)
RLC
p-NMHC IIA
(S1943)
NMHC IIA
GAPDH

Fig 4B

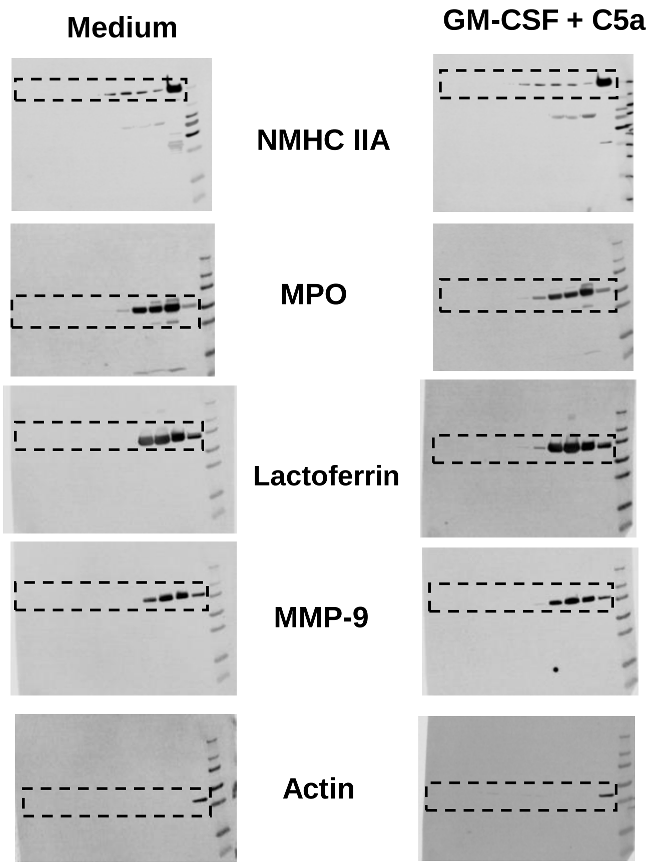

Medium
GM-CSF + C5a
NMHC IIA
MPO
Lactoferrin
MMP-9
Actin

Fig 4G

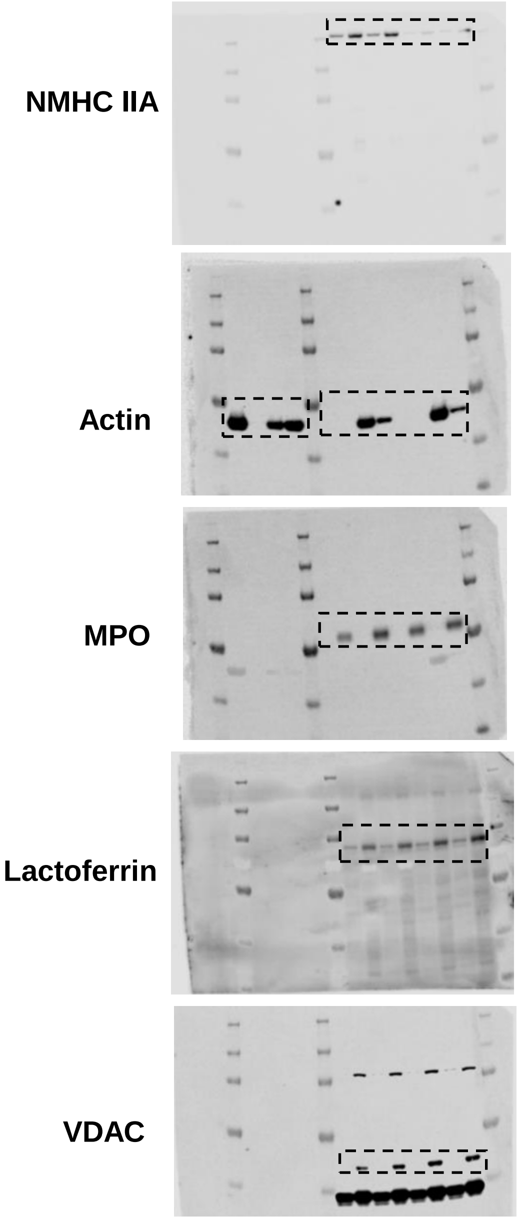
NMHC IIA
Actin
MPO
Lactoferrin
VDAC

Fig 4E

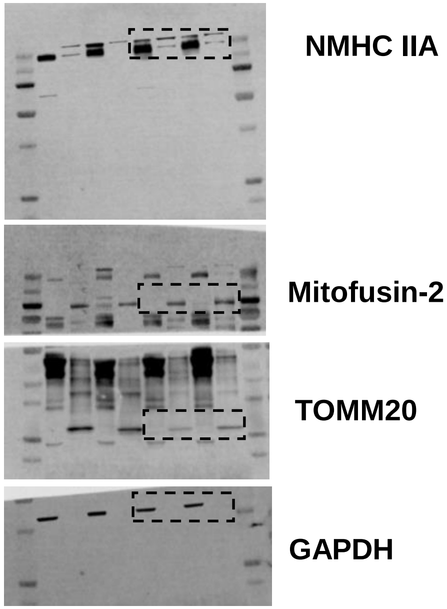
NMHC IIA
Mitofusin-2
TOMM20
GAPDH

Fig 5A

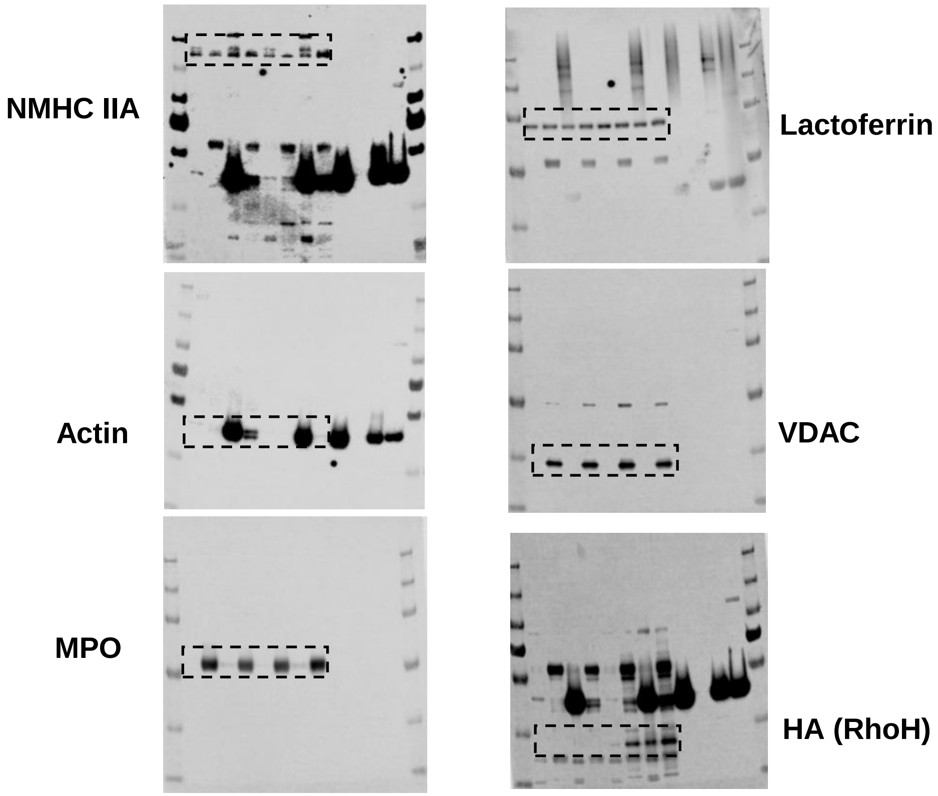
NMHC IIA
Lactoferrin
Actin
VDAC
MPO
HA (RhoH)

Fig 5C

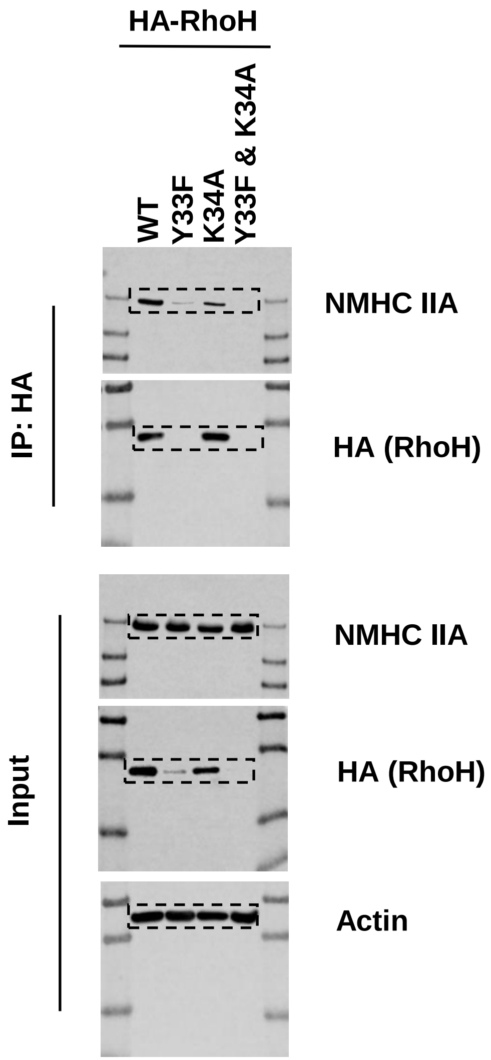
HA-RhoH
WT
Y33F
K34A
Y33F & K34A
IP: HA
NMHC IIA
HA (RhoH)
Input
NMHC IIA
HA (RhoH)
Actin

Fig 5D

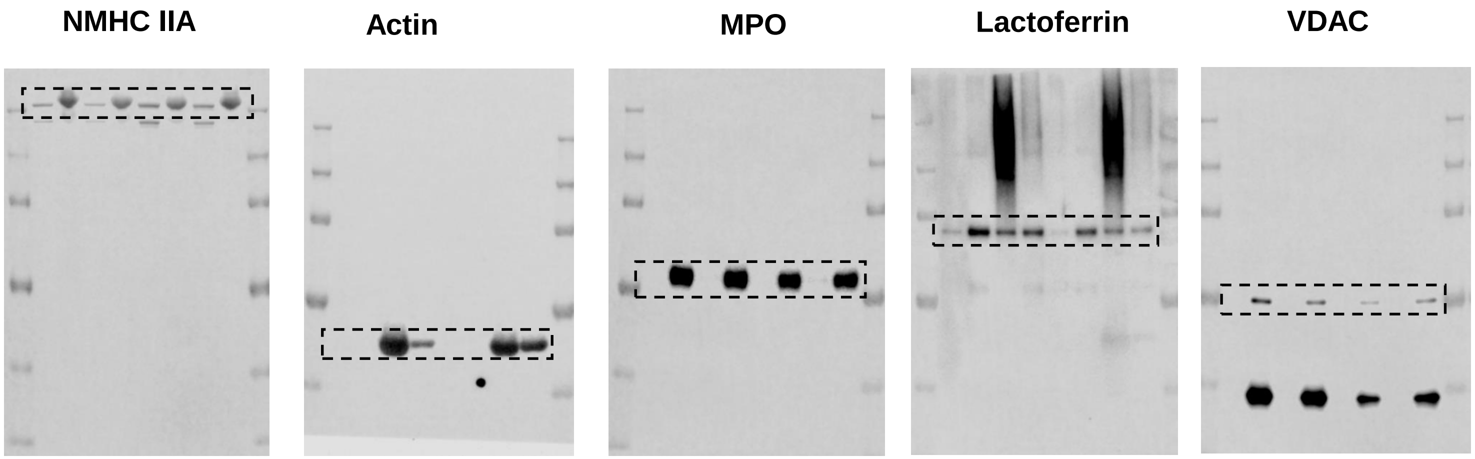

Fig 6B

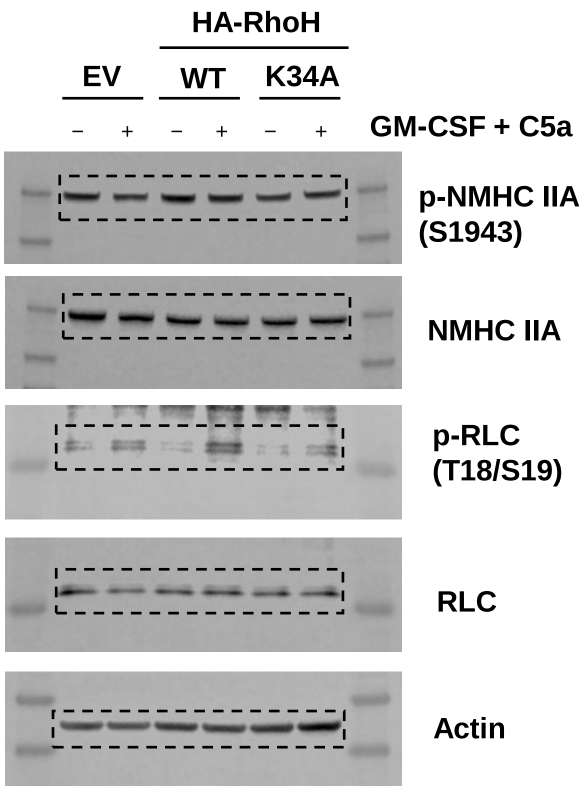

Fig 6C

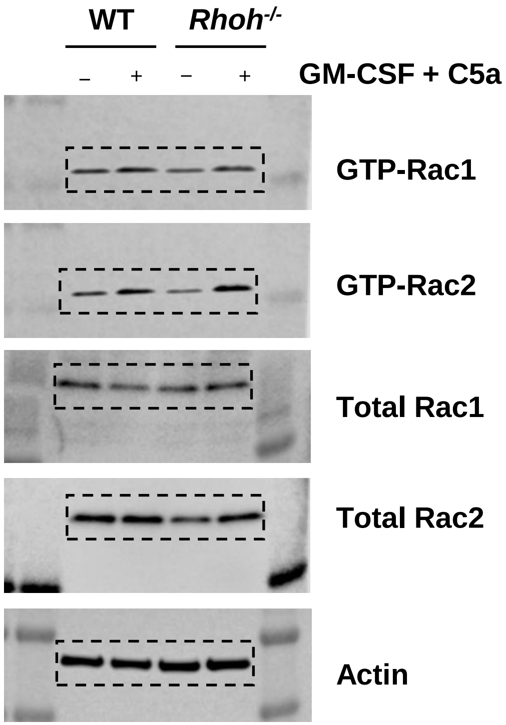

Fig 6D

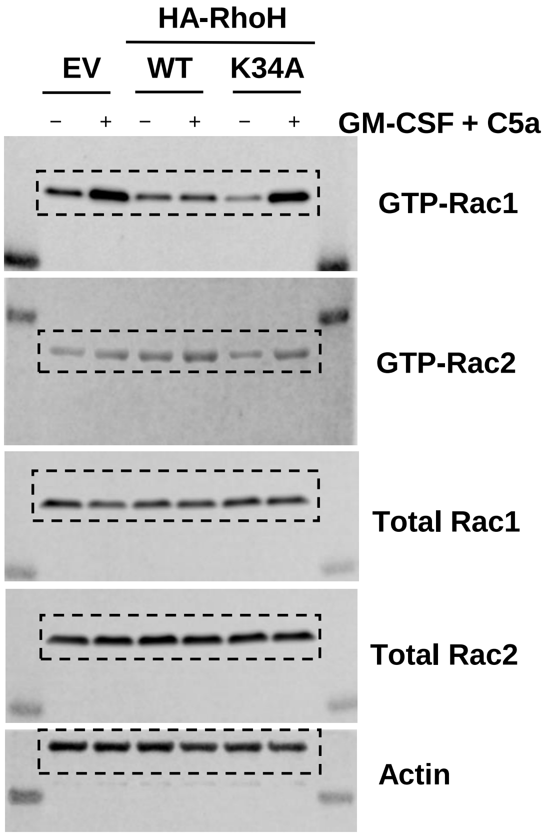

Fig 7A

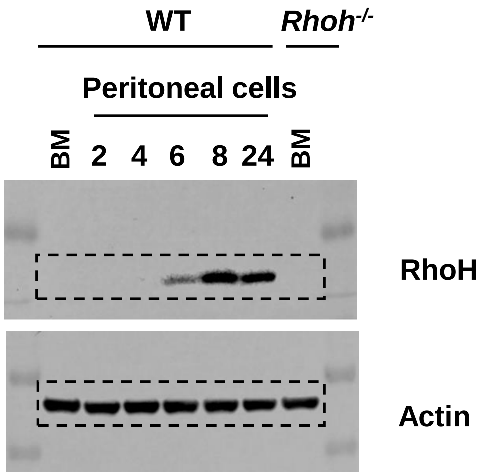
WT
Rhoh-/-
Peritoneal cells
BM
2
4
6
8
24
BM
RhoH
Actin

Fig 7H

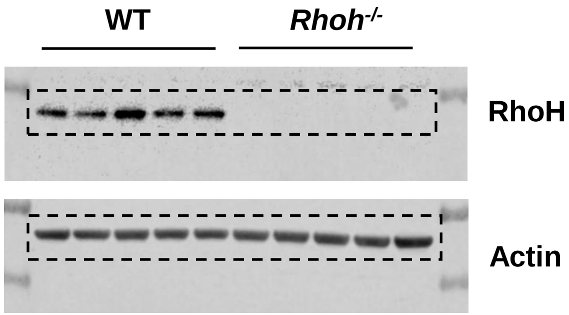
WT
Rhoh-/-
RhoH
Actin

S2A Fig

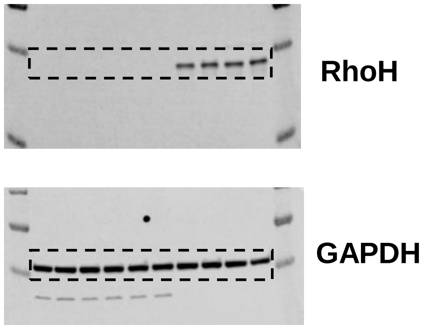

S5A Fig

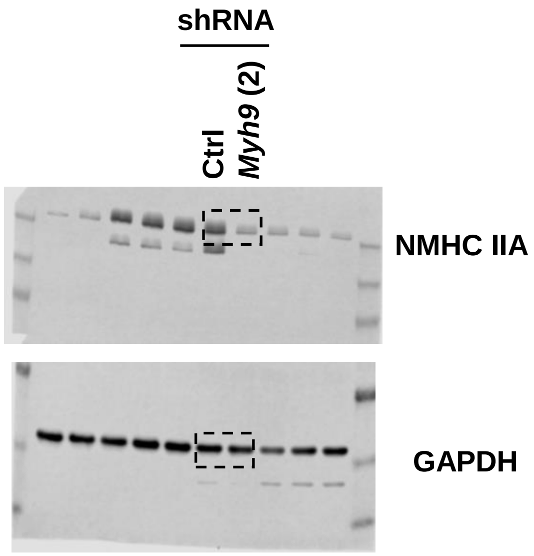

S6B Fig

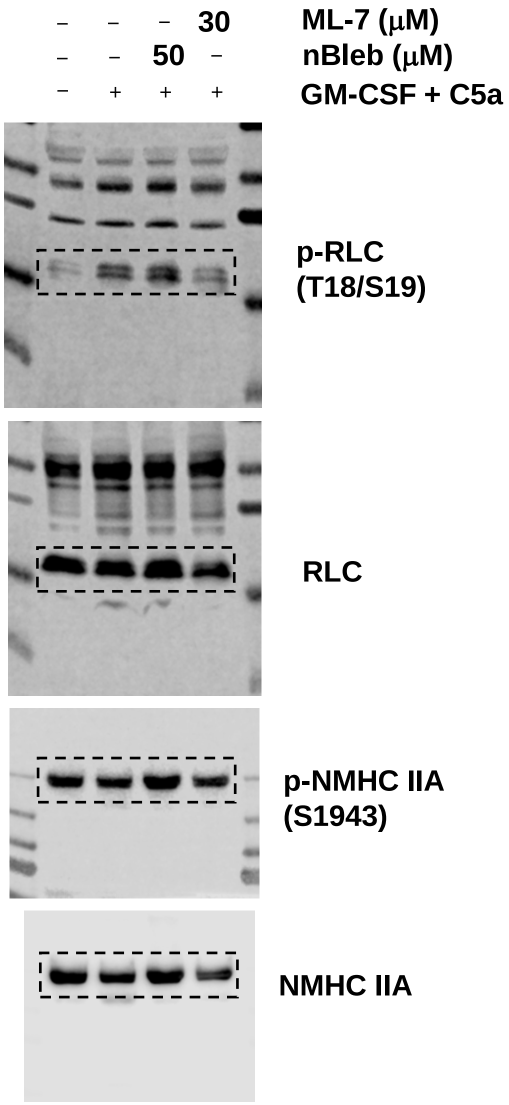

– – – 30 ML-7 (μM)
– – 50 – nBleb (μM)
– + + + GM-CSF + C5a
p-RLC (T18/S19)
RLC
p-NMHC IIA (S1943)
NMHC IIA

S7A Fig

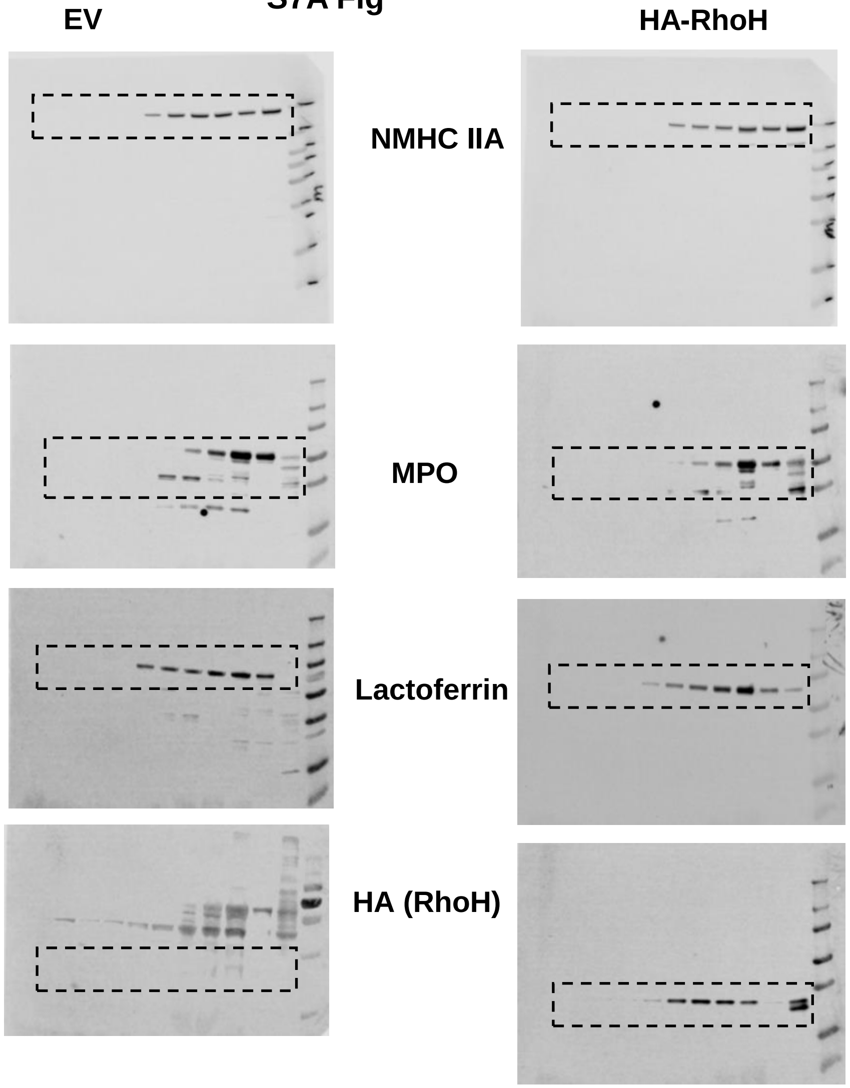

S7B Fig

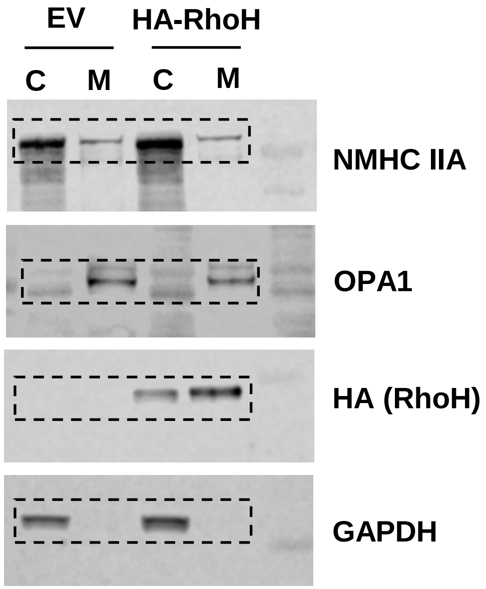

S8B Fig

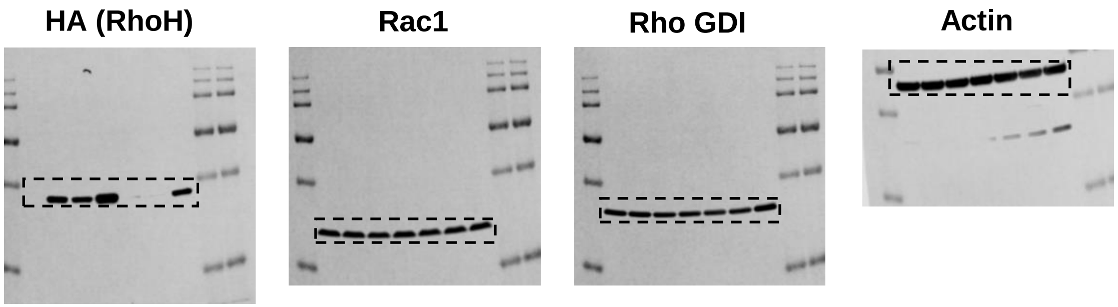

S9C Fig

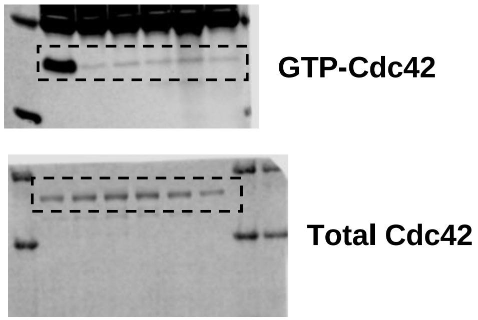

S9D Fig

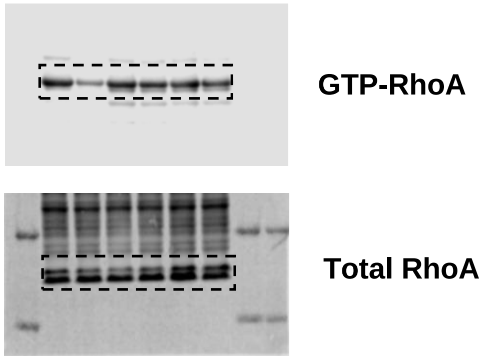

Supplement: S1 Raw images — (PDF) [file pbio.3001794.s011.pdf]
