# Supplementary material for: Nascent RHOH acts as a molecular brake on actomyosin-mediated effector functions of inflammatory neutrophils
Source: PLoS Biol. 2022 Sep 15;20(9):e3001794. doi: 10.1371/journal.pbio.3001794 (PMC9514642; doi:10.1371/journal.pbio.3001794)
Supplement: Attachment — Submitted filename: Point by point reply to reviewers and editor_rev3_final.docx [file pbio.3001794.s012.docx]

**Response to Reviewers**

We are grateful to the Reviewers for their valuable suggestions in the first round as well as their further comments. Our point-by-point responses to the comments are denoted below in blue. Major changes are indicated in red in the manuscript.

**Point-by-point response**

**Reviewer #1:** The original manuscript was very good, and the authors have addressed my comments in the revised version. They have toned down/removed previous overinterpretations, and I am very pleased that they have menasured Rac activity (new Figure 6). This showed that RhoH indeed negatively impacts on Rac1 activity, at least in a context of overexpression. I suspect that the RhoH deficient cells would also show a difference, with only slight tweaking of conditions, but I won't insist on that. I would also have preferred to see the Rac activity blots quantified by densitometry, but that alone does not warrant another round of revision. If other reviewers ask for a second round, please include this minor point.

We agree with the Reviewer that increased Rac1 activity might be detectable in RhoH-deficient cells by optimizing the experiment conditions, such as the stimuli used and the time for treatment. We are interested in testing these possibilities in our future work.

In the revised manuscript, the Rac activity blots were quantified and the statistics in a graph format have been added to Figure 6C, D.

**Reviewer #3:** The authors have addressed most of my points with additional data and/or changes to the text.

Major concern: The new data provided (specifically the list of HA-RhoH interactors provided just for the reviewer) highlight that the interaction between RhoH and NMHCIIA is unlikely to be specific, as I stated in my original point 7. All of the proteins in the table are abundant and frequently detected by mass spectrometry in protein immunoprecipitations. Usually they are removed by proteomic bioinformaticians because they are known to be frequent contaminants, including myosins. I appreciate that the authors have some selectivity of RhoH/NMHCIIA association based on subsequent immunoprecipitations. Annexin-A1 is an equally plausible HA-RhoH 'interactor' based on the peptide coverage. Notably, a bioID interactome for RhoH did not detect NMHCIIA as an interactor (albeit in Hela and HEK293 cells, but these both express NMHCIIA). See Nature Cell Biol 2019, Cote JF (corresponding author).

We agree with the Reviewer that proteins like myosins are frequently detected by mass spectrometry due to their high abundance. They are considered as contaminants under some conditions, but not always the case, depending on the context. For instance, NMHC IIA (MYH9) has been identified as the interactor of small molecule J13 in HepG2 cells (Figure 1F, G; PMID: 34386324), HBX protein in HCC cells (Figure 4a, b; PMID: 32296025), as well as NEK9 protein in MEFs (Figure 5a, b; PMID: 34078910), respectively. Therefore, cautions should be taken when removing these proteins.

Based on the subsequent immunoprecipitation, Annexin A1 did not show selectivity in interaction with RhoH between control and GM-CSF plus C5a-treated cells, while NMHC IIA did (please see below the representative immunoblot data). For this reason, we focused the remaining studies on NMHC IIA.

The BioID interactome is indeed a useful approach to define the protein interaction network, and a number of candidate interactors of RhoH have been detected in the context of HEK293 and HeLa cells, albeit without experimental validation. However, one important aspect to keep in mind is that the proximity interactions might be reshuffled when cells integrate various stimuli. For instance, in the study that proves the association between RhoH and Zap70 (PMID: 17028588), the author observed the interaction was enhanced more than 20-fold when constitutively active Lck was co-expressed with RhoH and Zap70 in HEK293 cells. Similarly, TCR engagement significantly increased the interaction of RhoH with Zap70. Moreover, RhoH can be post-translationally modified and play roles in a cell-type specific manner, as stated in the manuscript (page 3, 4, 18). These aspects may explain why NMHC IIA was not detected as a potent interactor of RhoH in HEK293 and HeLa cells.

Note that some of my previous points have not been addressed:

We appreciate the reminder from the Reviewer and apologize for the unaddressed points.

1. I said that both the abstract and the introduction need to state that 'RhoH is constitutively GTP-bound'. The abstract still needs re-wording.

We have now corrected this point (page 2).

5. Please state specifically in the Introduction that RhoH protein was found to be upregulated by after CF treatment, and that RhoH mRNA was found to be upregulated by GM-CSF, as explained in their reply to my question.

We have now modified the text following the Reviewer’s suggestions (page 4).

11. Thank you for adding the cell spread area without GM-CSF. As expected based on my comments, blebbistatin alone induces cell spreading. Therefore it is not surprising that there is no additional effect of GM-CSF, because the cells are already spread. The text needs to be clear on this point. The p value for the difference of control cell spread area +/-blebbistatin should be included.

We have now stated in the text “Subsequent activation of these cells by GM-CSF plus C5a did not induce significant cell spreading additionally” (page 10).

Please note that the requested P value was already included in the previous round of revision. We now add the P value between blebbistatin-treated control and activated cells to support that there was no additional effect of GM-CSF and C5a on cell spreading (Figure S6A).

11. 'How many cells were analyzed in total and how many per experiment for the measurements of F-actin fluorescence?'. The reply says that Darko would provide these data but unfortunately the information is still missing from the figure legend.

We apologize for the missing information. We have now provided such information in the figure legends (page 45 and 53).

13. The reply states 'Quantification has been performed and statistics were added (Figure 6C and 6D).' The quantification for the individual experiment is indicated, but there are no statistics (there should be results from 3 independent repeats quantified in a graph next to Figure 6C and Figure 6D). Also, I note that Rac2 is in fact activated by GM-CSF/C5a (based on the quantification of the single experiment shown). Quantification of multiple experiments is therefore essential, to prove whether this is reproducible or not.

Statistics from three independent repeats have been provided in a graph format and added underneath the blots (Figure 6C and 6D).

14. The reply states' We agree with the Reviewer's point that the molecular docking analysis can only suggest potential interaction sites.' The text still says 'show' so needs changing. In addition, Supporting Figure 2D (alignment of RhoH with Rac1, Cdc42 etc.) is useful and should be added to the paper figures, as well as mentioned in the text.

We thank the Reviewer for pointing this out and have corrected “Molecular docking analysis suggests that…” (page 12).

As suggested by the Reviewer, the alignment of RhoH with the classical RHO GTPases has been added to Figure 5B and described in the text (page 13).

15. Quantification of western blots: Figure 6D has not been quantified, even though it is in the authors' list of 'quantified blots', and needs to be done. Rac1/Rac2 activity assays can be very variable between experiments, hence the need to show reproducibility.

The requested quantification and statistics are now added to Figure 6D.

**Response to Editors**

We thank the PLOS Biology editorial team and the academic editor for their feedback and suggestions. Please find below our response (indicated in blue) to each request. Changes are denoted in red in the revised manuscript.

**Point-by-point response**

Whilst we appreciate that you have provided quantifications for the western blots presented in Figure 6, we ask that you please provide the quantifications and statistics in a graph format so that they are easier for the reader to spot.

Done (Figure 6B, C and D; S1 Data).

Please also make sure to address the following data and other policy-related requests that I have provided below (A-G):

(A) In the ethics statement provided in the manuscript:

- Please move the ethics statement to the Methods section (‘Mice’ subsection)

Done (page 21).

- Please provide the specific approval number issued by the Cantonal Veterinary Office of Bern and confirm whether the Veterinary office is an IACUC/ethics committee that consists of a panel of experts that reviewed and approved the study. In addition, please provide the method of euthanasia used in the experiments.

Done (page 21).

- Please provide the specific approval number issued by the Ethics committee of Canton Bern for the human subject’s research.

Done (page 22).

(B) Thank you very much for already providing the underlying data for the Figures presented in the manuscript. This looks complete, but I note that the underlying data for Figure S1B-C (GEO datasets) has not been included. I would be grateful if you could add this data in the source data file.

Thank you for this point. Please note that Figure S1B-C were produced by reanalyzing publicly accessible single-cell RNA sequencing (scRNA-seq) datasets. Due to the big size of the datasets and the involvement of the third-party data use, we did not include these data in the source data file. To address this, we have cited the original paper and indicated the accession number used to identify the datasets in the manuscript (page 5). In addition, we now provide information for the underlying data in the figure legend:

S1B. Fig: The underlying data can be found at <https://www.ncbi.nlm.nih.gov/geo/query/acc.cgi?acc=GSE158055>

S1C. Fig: The underlying data can be found at <https://www.ebi.ac.uk/biostudies/arrayexpress/studies/E-MTAB-8832>

(C) For figures containing FACS data, we ask that you please submit the raw FCS files to the FlowRepository (<https://flowrepository.org/>). We also ask that this data be made publicly available in the repository before publication.

The raw FCS files have been made public and are now accessible via:

<http://flowrepository.org/id/FR-FCM-Z5MZ>

<http://flowrepository.org/id/FR-FCM-Z5LV>

<http://flowrepository.org/id/FR-FCM-Z5M3>

<http://flowrepository.org/id/FR-FCM-Z5MY>

<http://flowrepository.org/id/FR-FCM-Z5M2>

(D) Please also ensure that each of the relevant figure legends in your manuscript include information on *WHERE THE UNDERLYING DATA CAN BE FOUND*, and ensure your supplemental data file/s has a legend.

Done.

(E) In addition, thank you for already providing the original and uncropped images of the blots presented in the manuscript. However, I note that the images are not fully uncropped and do not show the wider area of the gel surrounding the bands. If you have cut the blot membranes during the analysis and do not have access to the whole gel, then this is OK. If you happen to have the wider image, then we ask that you please replace and include in the file.

Done.

(F) Please ensure that your Data Statement in the submission system accurately describes where your data can be found and is in final format, as it will be published as written there. This would include providing the accession number/URL for any deposition at the FlowRepository.

Data Statement as follows:

The re-analyzed public accessible single-cell RNA sequencing (scRNA-seq) datasets can be found at: <https://www.ncbi.nlm.nih.gov/geo/query/acc.cgi?acc=GSE158055>;

<https://www.ebi.ac.uk/biostudies/arrayexpress/studies/E-MTAB-8832>.

The raw FCS files are available at the FlowRepository (<https://flowrepository.org/>) under ID: FR-FCM-Z5MZ, FR-FCM-Z5LV, FR-FCM-Z5M3, FR-FCM-Z5MY, FR-FCM-Z5M2. Other data can be found in the supporting information files.

(G) Please also provide a blurb which (if accepted) will be included in our weekly and monthly Electronic Table of Contents, sent out to readers of PLOS Biology, and may be used to promote your article in social media. The blurb should be about 30-40 words long and is subject to editorial changes. It should, without exaggeration, entice people to read your manuscript. It should not be redundant with the title and should not contain acronyms or abbreviations. For examples, view our author guidelines: https://journals.plos.org/plosbiology/s/revising-your-manuscript#loc-blurb

Here is our suggestion for the blurb:

Can neutrophils calm down during inflammation? A novel insight into the intracellular mechanisms how neutrophils control the strength of degranulation and neutrophil extracellular trap formation.

As you address these items, please take this last chance to review your reference list to ensure that it is complete and correct. If you have cited papers that have been retracted, please include the rationale for doing so in the manuscript text, or remove these references and replace them with relevant current references. Any changes to the reference list should be mentioned in the cover letter that accompanies your revised manuscript.

We added the missing page information for reference 3, 14 and 61.
